# Effects of psychological interventions on anxiety and pain in patients undergoing major elective abdominal surgery: a systematic review

**DOI:** 10.1186/s13741-020-00169-x

**Published:** 2020-12-08

**Authors:** Gianluca Villa, Iacopo Lanini, Timothy Amass, Vittorio Bocciero, Caterina Scirè Calabrisotto, Cosimo Chelazzi, Stefano Romagnoli, A. Raffaele De Gaudio, Rosapia Lauro Grotto

**Affiliations:** 1 Department of Health Sciences, Section of Anaesthesiology, Intensive Care and Pain Medicine, University of Florence, Largo Brambilla 3, 50100 Florence, Italy; 2 Department of Anaesthesia and intensive Care, Azienda Ospedaliero Universitaria Careggi, Florence, Italy; 3 Department of Medicine, Division of Pulmonary Sciences and Critical Care Medicine, University of Colorado, Denver, CO USA; 4 Department of Health Sciences, Section of Psychology and Psychiatry, University of Florence, Largo Brambilla 3, 50100 Florence, Italy

**Keywords:** Metabolic stress response, Perioperative care, Cognitive behaviour therapy, Mindfulness, Hypnosis, Narrative medicine

## Abstract

A maladaptive response to surgical stress might lead to postoperative complications. A multidisciplinary approach aimed at controlling the surgical stress response may reduce procedural complications and improve patients’ quality of life in the short and long term. Several studies suggest that psychological interventions may interact with the pathophysiology of surgical stress response, potentially influencing wound repair, innate and adaptive immunity, inflammation, perception of pain, and patients’ mood. The aim of this systematic review is to summarise the effects of perioperative psychological interventions on surgical pain and/or anxiety in adult patients scheduled for elective general abdominal and/or urologic surgery.

We conducted a systematic review of controlled clinical trials and observational studies involving psychological interventions for adult patients scheduled for elective general abdominal and/or urologic surgery. Only studies reporting pain and/or anxiety among outcome measures were included in the systematic review. The following psychological interventions were considered: (1) relaxation techniques, (2) cognitive-behavioural therapies, (3) mindfulness, (4) narrative medicine, (5) hypnosis and (6) coping strategies.

We examined 2174 papers. Among these, 9 studies were considered eligible for inclusion in this systematic review (1126 patients cumulatively): 8 are randomised controlled trials and 1 is an observational prospective pre/post study.

Psychological characteristics widely influence the pathophysiological mechanisms underlying the neuroendocrine and inflammatory response to surgical stress, potentially interfering with surgical outcomes. Psychological interventions are technically feasible and realistically applicable perioperatively during abdominal and/or urologic surgery; they influence the pathophysiological mechanisms underlying maladaptive surgical stress response and might have positive effects on patients’ surgical outcomes, such as pain and anxiety.

## Background

The optimisation of perioperative care may reduce postoperative complications and undesirable sequelae of surgery such as pain, fatigue, depression, and prolonged convalescence (Kehlet and Ph 2000). Current approaches based on multimodal and multidisciplinary interventions have proved to be the most adequate strategy to control the perioperative surgical stress response, reduce complications, and improve postoperative quality of life in the short- and long-term (Visioni et al. 2018). In this regard, the implementation of perioperative psychological interventions has been demonstrated effective in modulating the surgical stress response and improving outcomes in surgical patients, particularly in those with maladaptive psychological features (Nelson et al. 2013).

Non-physical, preoperative patient factors have emerged as strong predictors of surgical outcomes (Ellis et al. 2012; Rosenberger et al. 2006; Theunissen et al. 2012). In particular, anxiety, depression, and catastrophising attitudes have been significantly associated with postoperative complications (Arpino et al. 2004; Granot and Ferber 2005; Munafò and Stevenson 2001; Rainville et al. 2005; Walburn et al. 2009), impaired postoperative recovery, and increased re-hospitalisation rate (Rosenberger et al. 2006). The pathophysiological link between psychological/psychosocial factors and surgical outcomes has been widely recognised (Nelson et al. 2013). For example, patient behaviours (e.g. obesity, smoking, alcohol intake) and negative psychological states can both affect surgical recovery (Mavros et al. 2011). Furthermore, non-physical, preoperative patient factors may directly influence the neuroendocrine and inflammatory response to surgical stress (Mavros et al. 2011), thus affecting perioperative immune function and surgical outcomes.

Evidence of the impact of psychological factors on the pathophysiology of several diseases has led to the development of a wide range of interventions aimed at facilitating the mind’s capacity to influence physical health (Nelson et al. 2013; Wolsko et al. 2004). Psychological interventions might have a positive effect on the patient’s preoperative perception of emotions, cognitions, and behaviours and, as a consequence, on surgical outcomes. In this context, cognitive-behavioural techniques and other interventions, such as relaxation, mindfulness-oriented tasks, support to adaptive coping strategies or hypnosis, as well as supportive care and narrative medicine-based interventions, have all been identified as effective perioperative approaches to improve surgical stress response and outcomes. Such interventions can realistically be adopted in perioperative care and surgical procedures, and should be considered a feasible option to improve clinical practice (Johnston and Vögele 1993; Powell et al. 2016).

The large number of systematic reviews and meta-analyses focusing on the effects of psychological interventions on surgical outcomes in breast, cardiac, or orthopedic surgery patients suggest this kind of approaches be most frequently contemplated in these specific settings (Richards et al. 2017; Szeverenyi et al. 2018). In other surgical specialities (even abdominal surgery, which includes some of the most common surgical procedures worldwide), a less systematic approach to evaluation of the effects of psychological interventions on surgical outcomes seems to have been adopted.

With these concepts in mind, the aim of this systematic review is to analyse the effects of the most common psychological interventions on surgical pain and/or anxiety in adult patients scheduled for elective general abdominal surgery.

## Methods

A systematic review was carried out in accordance with PRISMA guidelines (Moher et al. 2009). We considered only prospective, controlled clinical trials and observational studies involving psychological interventions in adult patients scheduled for elective general abdominal and/or urologic surgery. Only studies reporting pain and/or anxiety among outcome measures were included in the systematic review. Minimum follow-up time was not considered mandatory. The analysis has been confined to those psychological interventions considered to be realistically applicable during perioperative management of abdominal surgery patients. Specifically, the following psychological interventions were considered: (1) relaxation techniques, (2) cognitive-behavioural therapies, (3) mindfulness, (4) narrative medicine, (5) hypnosis, (6) coping strategies (see Table 1 for definitions).

**Table 1:** Definitions of Psychological perioperative treatments considered in this study

***Psychological perioperative treatments***	
*Cognitive-behavioural therapies* Psychosocial interventions aimed at identifying and challenging maladaptive thoughts, positively modifying feelings and behaviours, and thereby experiences; interventions may focus on the cognitive component or directly influence behavioural responses (Rolving et al. 2014)*.*	
*Relaxation techniques* Physical and cognitive treatments (such as progressive muscle relaxation, simple relaxation, breathing practices, music relaxation) aimed at reducing sympathetic arousal, increasing the feeling of calm, and improving self-control (LaMontagne et al. 2003; Michie et al. 2008).	
*Mindfulness-based interventions* Psychological interventions inspired by religion-based practices of meditation and contemplation; these presuppose patient engagement in the relevant aspects of the present experience in a non-judgmental manner (Kaplan et al. 1993).	
*Coping strategies* Behavioural and psychological strategies employed to master, tolerate, reduce, or minimise stressful events.	
*Hypnosis* Cognitive-behavioural technique with no specific side effects used to encourage and evaluate responses to suggestions (Hızlı et al. 2015).	
*Narrative medicine* Medical approach that acknowledges the value of people’s narratives and individual stories, focusing on the relational and psychological dimensions that are implied in physical illness.	

### Search methods and data extraction

**Fig 1.**
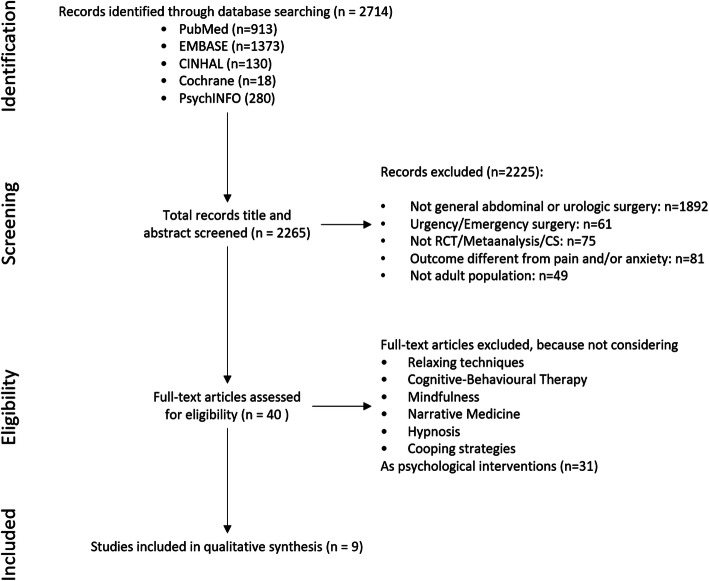
Flow chart of the screening process used to identify eligible studies. Exclusion criteria are not mutually exclusive and identified studies can fall into more than one category

A computerised search was performed in the following electronic databases: the Cochrane Register of Controlled Trials, PubMed, EMBASE, PsycINFO, and CINAHL. In order to limit confounding factors and ambiguous definitions, we limited the literature review to studies published in English from January 2000 to December 2019. The following search terms were used: ‘abdominal surgery’, ‘urologic surgery’, ‘cognitive behavioural therapy’, ‘relaxation therapy’, ‘mindfulness’, ‘coping’, ‘hypnosis’, ’narrative medicine’, ’psychological intervention’, ’pain’, and ’anxiety’. Strings used for searching the databases are available as supplementary material. The categories and assumptions underpinning the screening process are shown in Fig. 1.

Two authors (VB and GV) performed the records screening (title and abstract) by independently cross-checking data from identified studies with inclusion/exclusion criteria. A third author (IL) resolved the conflicts. The following data were extracted from the selected papers using a uniform spreadsheet: study identifiers, methods, examined interventions, and surgical outcomes.

## Results

A flow chart of the selection process used to identify eligible studies is shown in Fig. 1. Of the nine studies eligible for inclusion in the present review, eight are randomised controlled trials (Broadbent et al. 2012; Good et al. 2010; Hansen 2015; Hızlı et al. 2015; Lin and Wang 2005; Rejeh et al. 2013; Roykulcharoen et al. 2004; Zhang et al. 2013) and one is an observational prospective pre/post study (Sockalingam et al. 2019). Seven papers show data on abdominal surgery (Broadbent et al. 2012; Good et al. 2010; Lin and Wang 2005; Rejeh et al. 2013; Roykulcharoen et al. 2004; Zhang et al. 2013), while only one shows data on urologic surgery (Hızlı et al. 2015); one study presents data from a mixed population undergoing general abdominal and urologic surgery (Hansen 2015). A total of 1126 patients were included in the systematic review. Among them, 398 (35.3%) were male, while 728 (64.7%) were female; mean age was 52.9 years. Only 6 (Broadbent et al. 2012; Good et al. 2010; Hızlı et al. 2015; Roykulcharoen et al. 2004; Sockalingam et al. 2019; Zhang et al. 2013) out of 9 studies describe the percentage of patients undergoing oncological surgery. Among the 835 patients cumulatively described in these 6 studies, 303 (36.3%) underwent surgery for malignancy. The remaining three studies (Hansen 2015; Lin and Wang 2005; Rejeh et al. 2013) do not report the primary disease requiring surgical care.

Information on psychological treatments and surgical outcomes in the selected studies is reported in Table 2. We did not identify any study specifically aimed at evaluating the effect of mindfulness-based interventions or narrative medicine on perioperative anxiety and pain. Of the nine studies included in the review, five demonstrate a statistically significant reduction in perioperative pain due to psychological therapies (Good et al. 2010; Hızlı et al. 2015; Lin and Wang 2005; Rejeh et al. 2013; Roykulcharoen et al. 2004); in one paper, psychological therapies were not associated with an improvement in perioperative pain management (Hansen 2015). Six studies demonstrate a statistically significant reduction in perioperative anxiety due to psychological therapies (Broadbent et al. 2012; Hızlı et al. 2015; Lin and Wang 2005; Rejeh et al. 2013; Sockalingam et al. 2019; Zhang et al. 2013); in two papers, psychological therapies were not associated with an improvement in the management of perioperative anxiety (Hansen 2015; Roykulcharoen et al. 2004).

**Table 2:** Summary of the relevant surgical outcomes observed in the selected studies. Methods and scales used for outcome measurements appear in brackets

Years	First author	Sample size (pts)	Type of study	Type of intervention	Type of population	Relevant findings
2003	Roykulcharoen V et al. (2004)	102	RCT	Relaxation therapy	Abdominal surgery	The *relaxation* group had significantly less post-test sensation and distress of pain than the control group (VAS reduction: 56% vs 5%, *p* < 0.001); furthermore, the *relaxation* group had less anxiety (STAI reduction 9.6% vs 5.5%) or less than 6-h opioid intake (8.1 vs 7.5 mg), although without statistical significance.
2005	Lin L et al. (2005)	62	RCT	Coping strategies	Abdominal surgery	Compared to the control group, patients in the *coping* group experienced a significant decrease in preoperative anxiety (mean VASA: 3 vs 4.7, *p* < 0.001) and lower pain intensity (mean modified APSPO Questionnaire: 4.1 vs 5.1, *p* < 0.05) in the first postoperative day.
2010	Good M et al. (2010)	517	RCT	Relaxation therapy	Abdominal surgery	Perioperative *relaxation* significantly reduced pain in the first (effect in VAS reduction 24%, *p* = 0.001) and in the second (effect in VAS reduction 25%, *p* = 0.04) postoperative days.
2012	Broadbent E et al.(2012)	60	RCT	Relaxation therapy	Laparoscopic cholecystectomy	Lower anxiety and stress were observed in the *relaxation* group compared to the control group from pre-intervention to 7-day follow-up (mean PSS reduction 2.5 vs 0.5, *p* < 0.05).
2013	Zhang X et al. (2013)	60	RCT	Coping strategies and behavioural therapies	Oesophageal cancer	Compared to the control group, patients treated with *coping* and *behavioral therapies* had lower anxiety (SCL-90 score 1.6 vs 2, *p* < 0.05) and overall psychological distress (GSI 1.6 vs 1.8, *p* < 0.05).
2013	Rejeh N et al. (2013)	124	RCT	Relaxation therapy	Abdominal surgery	Compared to the control group, patients treated with *relaxation techniques* had lower pain (VAS: 1.9 vs 3.6, *p* < 0.001), anxiety (anxiety score: 2.5 vs 3.7, *p* < 0.001) and opioids requirements (*p* = 0.04).
2015	Hansen M et al. (2015)	105	RCT	Relaxation therapy	Abdominal and urological one day surgery	*Relaxation techniques* were not statistically associated with reduction in postoperative anxiety (STAI preop/postop: 2.2/3.3, *p* > 0.05) and pain (NRS preop/postop: 2.2/2.8, *p* > 0.05).
2015	Hizli F et al. (2015)	64	RCT	Hypnosis	TRUS-Guided Prostate Needle Biopsy	Compared to the control group, patients treated with *hypnosis* had significantly lower pain (VAS 1 vs 3, *p* = 0.011) and anxiety (BAI: 6 vs 2, *p* < 0.001, and HAS 11 vs 6, *p* < 0.001).
2019	Sockalingam S et al. (2019)	43	Observational prospective pre/post study	Cognitive behavioural therapies	Abdominal surgery	Tele-*cognitive behavioural therapies* delivered 1-year post-intervention improved psychological distress (PHQ-9 pre/post 12.4/9, *p* = 0.02) and anxiety (GAD-7 pre/post: 13.4/5.5, *p* < 0.005).

Abbreviations: *RCT* randomised controlled trial, *VAS* visual analogue scale, *STAI* Trait-State Anxiety Inventory scale, *VASA* visual analogue scale for anxiety, *APSPO* American Pain Society Patient Outcome, *PSS* Perceived Stress Scale, *SCL-90* Symptom Checklist-90, *GSI* Global Severity Index, *NRS* numeric rating scale, *TRUS* transrectal ultrasound, *BAI* Beck Anxiety Inventory, *HAS* Hamilton Anxiety Scale, *PHQ-9* Patient Health Questionnaire 9-Item scale, *GAD-7* Generalised Anxiety Disorder 7-Item scale

## Discussion

In this systematic review, we summarise the effects of perioperative psychological interventions (such as cognitive-behavioural therapies, relaxation techniques, mindfulness-based interventions, hypnosis, coping strategies, and narrative medicine) on surgical pain and/or anxiety in adult patients scheduled for elective general abdominal and/or urologic surgery. Several studies suggest that psychologic/psychosocial, preoperative patient factors directly interact with the pathophysiological mechanisms involved in the surgical stress response (Mavros et al. 2011), potentially influencing wound repair, innate and adaptive immunity, inflammation, perception of pain, and mood. Here, we describe how psychological interventions can influence pain and/or anxiety in abdominal surgery patients through interaction with the pathophysiological mechanisms underlying the neuroendocrine and inflammatory response to surgical stress.

Acute and/or chronic stress, including surgery-related perioperative stress, has been demonstrated to extensively affect patients’ neuroendocrine pathways (Maduka et al. 2015). Interestingly, there is evidence to suggest that psychological therapies might modulate perioperative neuroendocrine homeostasis in patients undergoing abdominal surgery (Manyande et al. 1995). On this basis, considering in particular the neuroendocrine effects of psychological interventions on endogenous opioid response and gate control system, Roykulcharoen and colleagues designed a randomised controlled trial aimed at demonstrating the positive effect of systematic relaxation on postoperative pain in abdominal surgical patients (Roykulcharoen et al. 2004). In this study, subjective (based on VAS scores) and objective (based on 6-h opioid intake) assessment of pain revealed that patients randomised to relaxation therapy experienced less postoperative pain (Roykulcharoen et al. 2004). Using a similar approach, Good and colleagues explored the effects of relaxation therapy in a randomised controlled trial with 517 abdominal surgery patients (Good et al. 2010). The rationale for this study is the ability of relaxation therapies to enhance a natural analgesic effect via increased parasympathetic activity and endogenous inhibitory mechanisms. In this study, psychological treatments were associated with a 25% reduction in VAS scores postoperatively (Good et al. 2010).

In line with these results, most of the psychological treatments examined in this review seem to be able to increase the secretion of inhibiting hypothalamic hormones, such as somatostatin or dopamine, and decrease the secretion of releasing hormones, such as thyrotropin- and corticotropin-releasing hormones and the growth hormone-releasing factor (Jindal et al. 2013). As a consequence, cortisol levels decrease (Walton et al. 1995) whereas levels of beta-endorphins may increase (Jindal et al. 2013). All these factors may contribute to improve pain experience and reduce anxiety associated with abdominal surgical procedures. Manyande and colleagues designed a controlled trial with 51 patients undergoing general abdominal surgery to test the capability of relaxation techniques and guided imagery to increase the feeling of being able to cope with surgical stress. This study showed that patients in the interventions group had less severe pain and less frequent postoperative complications than those in the control group. In addition, cortisol levels evaluated immediately before and after surgery were lower in patients receiving psychological treatments (Manyande et al. 1995).

Psychologic/psychosocial patient factors have been demonstrated to interact with perioperative inflammation (e.g. cytokine expression (Broadbent et al. 2003)), and thus with wound repair (Glaser et al. 1999) and pain perception. On this basis, considering in particular the effects of psychological stress on leptin resistance, neuropeptide Y and inflammatory cytokines, Sockalingam and colleagues designed an observational prospective pre/post study aimed at demonstrating the positive effect of perioperative cognitive behavioural therapy in a group of abdominal surgery obese patients undergoing bariatric surgery (Sockalingam et al. 2019). The authors demonstrated the positive effect of this type of psychological therapy on postoperative depressive symptoms, anxiety, and eating psychopathology (Sockalingam et al. 2019). In an observational study, Glaser and colleagues associated the symptoms of psychological stress with an ineffective regulatory pattern for IL-1 and IL-8 production in the wound site (Glaser et al. 1999). Similar results were found in an observational study of 47 adult patients undergoing surgical repair of inguinal hernia (Broadbent et al. 2003). The authors described the relationship between psychological stress and wound healing through the tissue levels of IL-1, IL-6, and matrix metalloproteinase-9. In the same study, preoperative psychological stress significantly predicted low levels of IL-1 and matrix metalloproteinase-9 in the surgical wound, as well as severe pain, postoperative complications, and poor and slow recovery (Broadbent et al. 2003). Consistent results were obtained by the same authors in a trial with 60 patients undergoing videolaparoscopic cholecystectomy randomised to treatment with relaxation therapies (Broadbent et al. 2012). Specifically, a greater reduction in anxiety and perceived stress was observed in the relaxation group compared with the control group from pre-intervention to 7-day follow-up (*p* < 0.05). Interestingly, the authors observed that patients treated with perioperative relaxation therapies had higher hydroxyproline deposition in the wound (i.e. expression of tissue repair) than those in the control group (difference in means 0.35, *p* = 0.03).

The interactions between psychological treatments and pathophysiology of surgical stress response might explain why these treatments have been associated with reduction of perioperative pain, anxiety, and pharmacological treatment requirements.

Hizli and others designed a randomised controlled trial including 64 patients scheduled for transrectal ultrasound-guided prostate needle biopsy to investigate the effects of hypnosis on pain and anxiety. Patients were randomised to receive a 10-min presurgery hypnosis session involving suggestions for increased relaxation and decreased anxiety. Post-intervention, and before surgery, patients in the hypnosis group had significantly lower mean values of pain and anxiety, measured using visual analogic scales, Beck Anxiety Inventory, and Hamilton Anxiety Scale, respectively (Hızlı et al. 2015). Similar results were obtained by Lin and colleagues from a randomised controlled trial involving 62 patients scheduled for abdominal surgical procedures. The authors found that preoperative coping procedures (such as nursing intervention for pain) had positive effects on preoperative pain anxiety, preoperative pain attitude, and pain perception (Lin and Wang 2005).

Interestingly, most of the studies identified in this systematic review mainly relied on subjective measures of pain, such as the visual-analogic or the numeric rating scales. Only two studies (Rejeh et al. 2013; Roykulcharoen et al. 2004) assessed the effects of psychological interventions on perioperative pain through objective measurements, e.g., analgesic requirements. Although both studies show consistent results on the effects of psychological therapies in reducing analgesic requirements, only in the randomised controlled trial by Rejeh et al. were these effects statistically significant (Rejeh et al. 2013).

As far as we are aware, this is the first systematic review focusing on the effects of psychological treatments on surgical pain and anxiety in patients undergoing abdominal and/or urologic surgery. Previous systematic reviews and metanalyses aimed at assessing the effects of psychological interventions in the context of orthopaedic or cardiac surgery. Results from our review are generally consistent with those obtained from studies involving orthopaedic or cardiac surgery patients. In particular, in a systematic review of 62 relevant studies published from January 1980 to September 2016, Szeverenyi and colleagues found that psychosocial interventions significantly reduced postoperative pain (Hedges’ *g* = 0.31 [95% CI = 0.14, 0.48]), and pre- and postoperative anxiety (*g* = 0.26 [0.11, 0.42] and *g* = 0.4 [0.21, 0.59], respectively), while no significant effects were associated to postoperative analgesic use (*g* = 0.16 [95%CI = 0.01, 0.32]. Similar findings were found by Rees and colleagues in a metanalysis exploring the effects of psychological treatments in patients with coronary artery diseases undergoing cardiac surgery. Analysing results from 36 trials including 12,841 patients, the authors showed a perioperative reduction in anxiety and depression. Notably, in both studies the authors included a higher number of studies compared with our review. That can be explained considering both the different temporal limits used for literature search and the historical interest among physicians in reducing symptoms related to the most painful surgical procedures, associated with a high incidence of severe postoperative pain and anxiety (e.g. orthopaedic surgery) or to conditions where anxiety and stress may affect patients outcomes (e.g. cardiac surgery). Moreover, differently from what was observed by Szeverenyi and colleagues, we observed a reduction in postoperative pain as well as a reduction in postoperative analgesic use in patients receiving psychological therapies.

The following limitations should be acknowledged. First, studies were limited to English language. Second, in one of the selected studies the effects of perioperative psychological interventions were studied in a cohort of bariatric patients. The peculiar psychological features of this population usually differ from those observed in the majority of abdominal and/or urologic surgical patients, potentially limiting the generalisation of the results. Third, in most studies perioperative pain was assessed through subjective scales (e.g. VAS or NRS). More objective indicators of intervention success (e.g. analgesic requirements) were used only in two of the identified studies.

Finally, the treatments explored and the outcomes observed in this review are different among the selected studies. For these reasons, a synthesis of the evidence on effectiveness of psychological treatments in reducing perioperative anxiety and pain has not been provided. Nonetheless, results seem to suggest a positive effect on anxiety and pain, that certainly merits further investigation in the abdominal/urologic setting.

## Conclusions

In conclusion, a maladaptive response to surgical stress negatively influences perioperative outcomes. Psychological characteristics widely influence the pathophysiological mechanisms underlying the neuroendocrine and inflammatory response to surgical stress, potentially interfering with wound repair, innate and adaptive immunity, inflammation, perception of pain, and mood. Interestingly, perioperative psychological interventions (such as cognitive-behavioural therapies, relaxation techniques, mindfulness-based interventions, hypnosis, coping strategies, and narrative Medicine) have proved effective on improving patients’ anxiety and pain, and have been shown to be technically feasible and realistically applicable perioperatively during abdominal and/or urologic surgery.

## Data Availability

The datasets of studies analysed during the current systematic review are available from the corresponding author on reasonable request.

## Abbreviations

IFN: Interferon
IL: Interleukin
NK: Natural killer
